# P-451. Evaluation of Febrile Infants with Bronchiolitis 29-60 days old

**DOI:** 10.1093/ofid/ofaf695.666

**Published:** 2026-01-11

**Authors:** Marilyn E Valentine, Jared Olson, Emily A Thorell, Anne J Blaschke

**Affiliations:** University of Utah, Salt Lake City, Utah; Intermountain Health, Salt Lake City, Utah; University of Utah, Salt Lake City, Utah; University of Utah School of Medicine, Salt Lake City, UT

## Abstract

**Background:**

Febrile infants with bronchiolitis have a low risk of bacterial infection. Current febrile infant algorithms require urine and blood testing, but the guidelines exclude infants with bronchiolitis. Urgent care and pediatrics clinics have the resources to provide initial evaluation of these infants' respiratory status, this my be a cost-effective strategy to reduce emergency department(ED) cost. We aimed to describe the initial evaluation of febrile infants 29-60 days old with possible bronchiolitis within our 23 hospital healthcare system.

Evaluation of Febrile Infants 29-60 days old with Bronchiolitis

**Figure:**
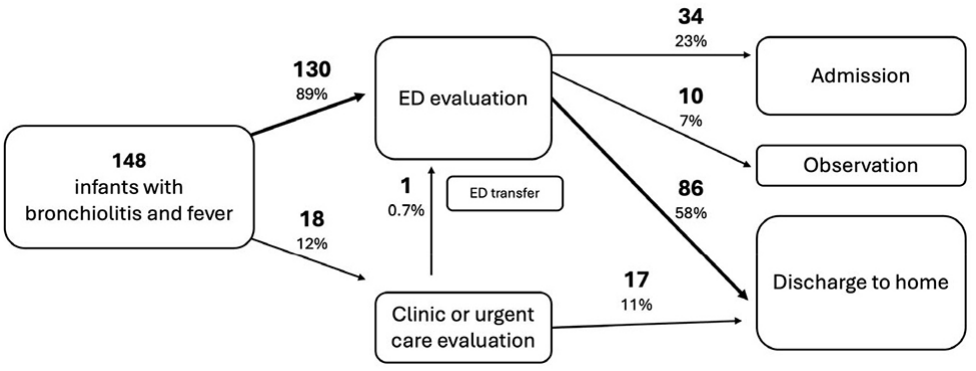

Location of Evaluation and Initial Disposition of Febrile Infants with Bronchiolitis

**Figure:**
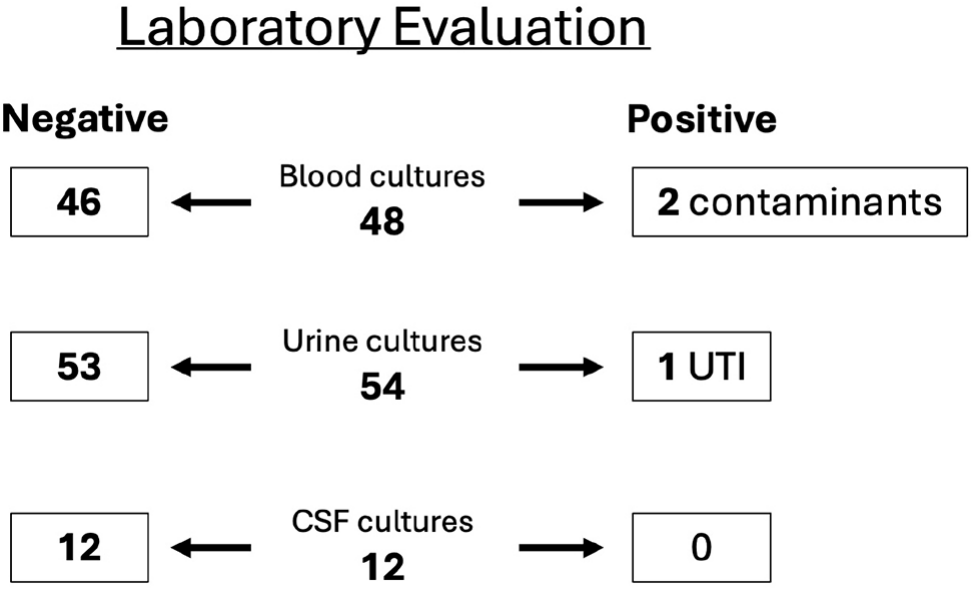

Blood, Urine and CSF cultures obtained on infants 20-60 days old with fever and bronchiolitis

**Methods:**

All febrile encounters in infants 29-60 days old in the Intermountain Healthcare system during 2024 were identified. A febrile episode was defined as fever in the chief complaint, problem list, or a measured temperature ≥38.0° C during the health care encounter. Infants with bronchiolitis were defined as bronchiolitis or congestion in the encounter problem list. We tracked ED visits and hospital transfers within 12 hours to determine severity of initial evaluation. We included cultures obtained on infants with bronchiolitis who underwent laboratory evaluation within 24 hours of their initial evaluation looking for significant bacterial infection (SBI).

**Results:**

We identified 628 infants aged 29-60 days with fever seen in 2024, 148 (24%) had possible bronchiolitis. Of the 130 (89%) infants evaluated in EDs, 48 (32%) had blood cultures with 2 positive for contaminants. 54 (36%) had urine cultures (1 positive) and 12 (8%) had CSF cultures (all negative), 44 (33%) were admitted or observed while awaiting cultures.The 18 (12%) patients evaluated in outpatient settings were seen in PCP clinics (9), or urgent cares (9). One required an ED transfer.

**Conclusion:**

Most febrile infants aged 29-60 days with bronchiolitis are currently managed in the ED, but could be managed in an outpatient setting. 70% of the febrile infants were not admitted or held for observation with only one infant identified with a SBI (urinary tract infection). 66% of the infants evaluated in the ED were not admitted or observed suggesting the diagnosis of bronchiolitis was not severe enough to warrant admission. A less costly outpatient evaluation for young febrile infants with non-severe respiratory symptoms may be sufficient.

**Disclosures:**

Anne J. Blaschke, MD, PhD, BioFire Diagnostics/Biomerieux: I have IP owned by the U. of Utah licensed to BioFire and receive royalties.|Merck & Co: Advisor/Consultant

